# Gastroprotection of Calein D against Ethanol-Induced Gastric Lesions in Mice: Role of Prostaglandins, Nitric Oxide and Sulfhydryls

**DOI:** 10.3390/molecules24030622

**Published:** 2019-02-11

**Authors:** María Elena Sánchez-Mendoza, Yaraset López-Lorenzo, Leticia Cruz-Antonio, Audifás-Salvador Matus-Meza, Yolanda Sánchez-Mendoza, Jesús Arrieta

**Affiliations:** 1 Escuela Superior de Medicina, Instituto Politécnico Nacional, Plan de San Luis y Díaz Mirón, Colonia Santo Tomás, Delegación Miguel Hidalgo, Ciudad de México 11340, Mexico; mesmendoza@hotmail.com (M.E.S.-M.); Hush_Puppies2050@live.com.mx (Y.L.-L.); 2 Facultad de Estudios Superiores Zaragoza, UNAM. Av. Guelatao No. 66, Colonia Ejército de Oriente Iztapalapa, Ciudad de México 09230, Mexico; letycruza@yahoo.com.mx; 3 Departamento de Farmacia, Facultad de Química, Universidad Nacional Autónoma de México, Ciudad de México 04510, Mexico; audi_matus@hotmail.com; 4 Unidad de Medicina Familiar No. 49, Instituto Mexicano del Seguro Social, Ciudad de México 06600, Mexico; samyola1@gmail.com

**Keywords:** calein D, gastroprotection, gastric ulcer, *Calea urticifolia*, sesquiterpene lactones

## Abstract

Peptic ulcers are currently treated with various drugs, all having serious side effects. The aim of this study was to evaluate the gastroprotective activity of calein D (from *Calea urticifolia*), a sesquiterpene lactone with a germacrane skeleton. Gastric lesions were induced in mice by administering ethanol (0.2 mL) after oral treatment with calein D at 3, 10 and 30 mg/kg, resulting in 13.15 ± 3.44%, 77.65 ± 7.38% and 95.76 ± 2.18% gastroprotection, respectively, to be compared with that of the control group. The effect found for 30 mg/kg of calein D was not reversed by pretreatment with N^G^-nitro-l-arginine methyl ester (l-NAME, 70 mg/kg, ip), indomethacin (10 mg/kg, sc) or *N*-ethylmaleimide (NEM, 10 mg/kg, sc). Hence, the mechanism of action of calein D does not involve NO, prostaglandins or sulfhydryl compounds. Calein D was more potent than carbenoxolone, the reference drug. The findings for the latter are in agreement with previous reports.

## 1. Introduction

Peptic ulcers are lesions in the gastric mucosa and duodenum that occur in more than 10% of the population worldwide. They develop as a result of an imbalance between defensive mechanisms (e.g., mucus, bicarbonate, prostaglandins, nitric oxide and sulfhydryl compounds) and aggressive factors. The latter consist of endogenous and exogenous agents, including hydrochloric acid, pepsin, *Helicobacter pylori*, nonsteroidal anti-inflammatory drugs (NSAIDs), alcohol abuse and stress [1,2].

The therapeutic strategy for peptic ulcers has focused mainly on eliminating pain, reducing gastric acidity and fortifying the gastric mucosal barrier [3]. Consequently, the current drugs for treating peptic ulcers are classified as antacids, antisecretory agents (histamine H_2_ receptor antagonists and proton-pump inhibitors) and gastroprotective drugs (sucralfate and misoprostol) [4]. However, none of them are completely effective and all cause serious side effects. For example, some recent studies have demonstrated that prolonged use of proton pump inhibitors, such as omeprazole, can lead to decreased absorption of vitamin B_12_, myocardial infarction and chronic kidney disease [5,6]. There is a need for new types of gastroprotective treatments.

One possible source of new drugs is the investigation of natural products, in particular medicinal plants [7]. In this sense, there is an important group of secondary metabolites, sesquiterpene lactones, which are found across some plants, most frequently occurring in the Asteraceae family. Of such compounds, the most common are germacranolides, guaianolides, pseudoguaianolides and eudesmanolides. Regarding gastroprotection, germacranolides are known to promote anti-ulcer and a wide range of other biological effects [8]. They contain an α-methylene-γ-lactone group that undergoes a Michael-addition reaction with free sulfhydryl or amino groups in proteins, which probably explains their gastroprotective activity [9].

Our group has described the gastroprotection produced by 2,3-epoxyjuanislamin [10] and calealactone B [11], two sesquiterpene lactones with a germacrane skeleton isolated from *Calea urticifolia*. Calein D, another sesquiterpene lactone from the same plant, is examined herein. Its gastroprotective activity was evaluated using the mouse model of ethanol-induced gastric lesions. Upon finding gastroprotection, the possible role of prostaglandins, nitric oxide and/or sulfhydryl groups was explored in their mechanism of action.

## 2. Results

### 2.1. Gastroprotective Activity of Calein D

**Figure 1:**
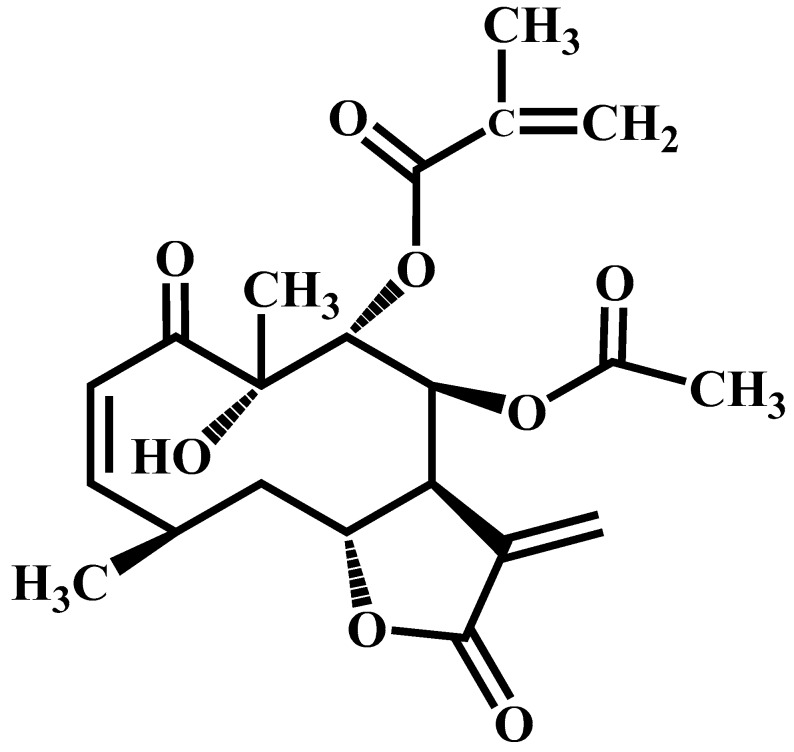
The structure of calein D.

**Figure 2:**
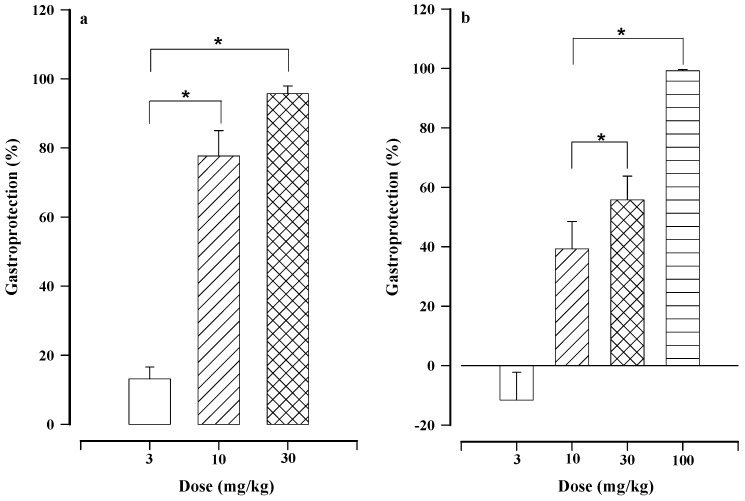
Gastroprotective effect of calein D (**a**) and carbenoxolone (**b**). Bars represent the mean ± SEM (*n* = 7). * *p* < 0.05, based on the Kruskal−Wallis test followed by Dunn’s multiple comparison.

Oral treatment with calein D (Figure 1) significantly reduced gastric lesions in a dose-dependent manner, resulting in the maximum effect at 30 mg/kg (95.76 ± 2.18% gastroprotection) and more limited activity at 10 and 3 mg/kg (77.65 ± 7.38 and 13.15 ± 3.44%, respectively, Figure 2a). This is the first report, to our knowledge, that calein D protects the gastric mucosa from damage caused by ethanol. Carbenoxolone also showed dose-dependent gastroprotection (Figure 2b), with a maximum effect (99.28 ± 0.38% gastroprotection) at the highest dose tested (100 mg/kg). As can be appreciated, the reference compound was less potent than calein D.

### 2.2. Effect of l-NAME, Indomethacin and NEM on the Gastroprotective Activity of Calein D

**Figure 3:**
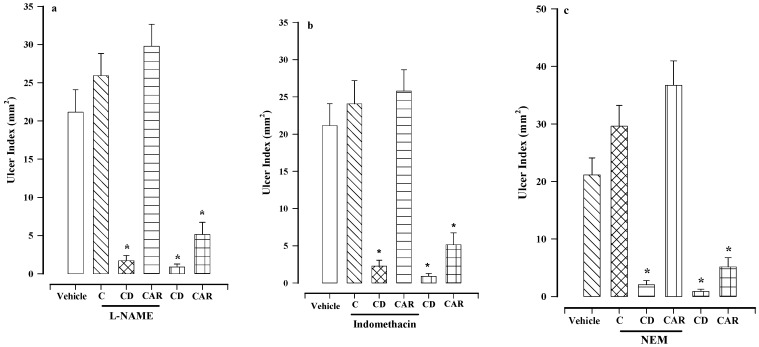
Effect of calein D (CD) and carbenoxolone (CAR) on gastric lesions induced by ethanol in mice pretreated with N^G^-nitro-l-arginine methyl ester (l-NAME) (70 mg/kg) (**a**), indomethacin (10 mg/kg) (**b**) or *N*-ethylmaleimide (NEM) (10 mg/kg) (**c**). C = the control group for the distinct inhibitors. Bars represent the mean ± SEM (*n* = 7). * *p* < 0.05 vs. the respective control, based on the Kruskal−Wallis test followed by Dunn’s multiple comparison.

The ulcer index was not significantly different when comparing control mice administered only Tween 80 at 0.05% (21.14 ± 2.84 mm^2^) and animals pretreated with 70 mg/kg of N^G^-nitro-l-arginine methyl ester(l-NAME) (25.93 ± 2.89 mm^2^, Figure 3a), 10 mg/kg of indomethacin (24.06 ± 3.12 mm^2^, Figure 3b) or 10 mg/kg of *N*-ethylmaleimide (NEM) (25.62 ± 2.59 mm^2^, Figure 3c). These results are in agreement with studies showing that the aforementioned inhibitors at the specified doses do not cause gastric damage [12].

The group receiving l-NAME (a nonspecific inhibitor of nitric oxide synthase) followed by calein D displayed an ulcer index of 1.71 ± 0.68 mm^2^, significantly lower than the value of the control group (21.14 ± 2.94 mm^2^). Thus, l-NAME did not alter the gastroprotective activity of calein D. On the other hand, the reversal of the effect of carbenoxolone by l-NAME pretreatment was in accordance with a previous report [10].

Pretreatment with indomethacin, a non-specific COX inhibitor, did not reverse the gastroprotective effect of calein D (Figure 3b), evidenced by the ulcer index of 2.25 ± 0.79 mm^2^, significantly different from the vehicle control group (Figure 3b). Contrarily, indomethacin pretreatment inhibited the effect of carbenoxolone, in agreement with the literature [10].

Pretreatment with NEM, a blocker of sulfhydryl groups, did not affect the mechanism of action of calein D (Figure 3c). The ulcer index of animals given NEM and then calein D was much lower (2.03 ± 0.7356 mm^2^) than the vehicle control group, suggesting that sulfhydryl groups are not involved in the mechanism of action. Regarding the reference drug, the present findings concur with previously published results [10].

## 3. Discussion

The classic treatment of gastric ulcers is based on antisecretory therapy, which gives rise to serious side effects in case of prolonged use [5]. Therefore, safer and more effective anti-ulcer agents are needed. The gastroprotective activity of calein D (obtained from *Calea urticifolia*) was presently evaluated with the mouse model of ethanol-induced gastric lesions [13]. Regarding the mechanism of action, the possible participation of prostaglandins, nitric oxide and sulfhydryl groups was explored.

Ethanol, often employed in a mouse model to provoke gastric ulcers, is known to damage the gastric mucosa by triggering the release of inflammatory mediators and increasing the levels of granulocytes, oxidizing metabolites, cytokines and vasoactive substances. In this context, ethanol promotes inflammation, vasoconstriction and ischemia [14]. Gastric lesions were herein induced by ethanol administration after oral treatment with calein D or the reference compound, carbenoxolone. Calein D significantly reduced the ulcer index, reaching the maximum effect at 30 mg/kg. The reference compound exhibited a maximum effect at 100 mg/kg, indicating the greater potency of calein D. Our group previously documented similar activity for 2,3-epoxyjuanislamin and calealactone B, two other sesquiterpene lactones with a germacrane skeleton isolated from *Calea urticifolia* [10,11]. 

The current finding confirms the importance of further research on sesquiterpene lactones with a germacrane skeleton in gastroprotection. In previous studies, calein D could not be isolated from *Calea urticifolia* because the plant did not produce the same secondary metabolites. Its content is dependent on seasonally-related environmental stress. The dose-dependent gastroprotective effect described for 2,3-epoxyjuanislamin and calealactone B was herein found for both calein D and carbenoxolone (Figure 2).

It has been demonstrated that nitric oxide, PEG_2_ and endogenous sulfhydryl groups protect the gastric mucosa against damage generated by ethanol. Hence, we examined the possible participation of these factors in the gastroprotective mechanism of action of calein D.

Nitric oxide plays an important role in many physiological processes, as shown by reports on its protective effect on the gastric mucosa by reducing motility, boosting mucus production and blood flow, and attenuating the inflammatory response [15]. Pretreatment with l-NAME, a nonspecific inhibitor of nitric oxide synthase, did not modify the protective effect of calein D (Figure 3a), meaning that mechanism of action of this compound does not involve the nitric oxide pathway. Contrarily, the effect of carbenoxolone was reversed by l-NAME, which is in agreement with the literature [10].

Prostaglandins, mainly PEG_2_, protect the gastric mucosa by activating its different EP receptors, thus enhancing the secretion of mucus and bicarbonate, increasing blood flow, and decreasing acid secretion [16]. Pretreatment with indomethacin, a non-specific COX inhibitor, did not reverse the gastroprotective effect of calein D (Figure 3b). Therefore, prostaglandins do not participate in the gastroprotection of calein D. Indomethacin pretreatment followed by carbenoxolone and gastric lesions led to the same results previously described [10].

Sulfhydryl groups such as glutathione protect the gastric mucosa by helping to maintain its structure and by eliminating free radicals [17]. Since pretreatment with NEM (a blocker of sulfhydryl groups) did not affect the mechanism of action of calein D (Figure 3c), the gastroprotective activity of the latter compound does not involve sulfhydryl groups. Regarding the reference drug, the findings are in accordance with the literature [10].

We have reported that neither prostaglandins, nitric oxide nor sulfhydryl groups participate in the mechanism of action of 2,3-epoxyjuanislamin or calealactone B [10,11]. According to Umemura et al. [18], both 2,3-epoxyjuanislamin and calealactone B can activate the Nrf2 system. Upon exposure to an oxidative stimulus, Nrf2 is known to translocate into the nucleus and bind to the antioxidant response element, which regulates the expression of antioxidant enzymes (e.g., CAT, SOD and GPx) involved in the protection of the gastric mucosa [19]. The activation of this system is triggered by the α,β-unsaturated carbonyl groups in the structure of these lactones, as they are susceptible to undergoing a Michael-type reaction [18,20] with the sulfhydryl groups of the cysteine residues of the proteins. On the other hand, proton-pump inhibitors like omeprazole are prodrugs that bind covalently to the sulfhydryl groups of cysteine residues in the extracellular domain of the ATPase H^+^/K^+^ through a Michael-type reaction to generate a stable disulfide complex, thereby inhibiting enzyme activity [21]. 

Hence, the α,β-unsaturated carbonyl groups in calein D may protect the gastric mucosa by activating the Nrf2 system and/or inhibiting ATPase H^+^/K^+^, hypotheses that should be explored in future research.

## 4. Material and Methods

### 4.1. Animals

Male CD1 mice (25–30 g) were provided by the animal house of the Universidad Autónoma Metropolitana, Xochimilco campus, Mexico City. All animal-related procedures were carried out in accordance with the Mexican Official Norm for Animal Care and Handling (NOM-062-ZOO-1999) and the international rules on the care and use of laboratory animals. The mice, individually housed in cages with wire-net floors, were deprived of food 18 h prior to experimentation. They were allowed free access to water throughout the procedures.

### 4.2. Drugs and Compounds

Carbenoxolone (the reference drug), N^G^-nitro-l-arginine methyl ester, *N*-ethylmaleimide and indomethacin were purchased from Sigma Chemical Co. (St. Louis, MO, USA). All substances were prepared minutes before use.

### 4.3. Isolation of Calein D

Six kilograms of *C. urticifolia* leaves were extracted by maceration during three days at room temperature (22 ± 2 °C), first with hexane (15 L × 3) and then with dichloromethane (15 L × 3). After evaporation of the solvents, 335 g of the dichloromethane extract was yielded. Subsequently, 300 g of this extract was subjected to silica gel column chromatography with hexane and mixtures of hexane/dichloromethane as the elution system. White crystals (2.1 g) were obtained from the fractions 134–148 (hexane/dichloromethane, 4:6). They were purified by recrystallization with a hexane/petroleum ether mixture (1:1) and identified as calein D (Figure 1) by comparing the ^1^H and ^13^C-NMR spectra to those reported in the literature [22].

### 4.4. Ethanol-Induced Gastric Ulcers

The procedure herein employed was a slightly modified version of that published by Al-Amin et al. (2012). Gastric ulcers were generated by oral administration of ethanol to mice (0.2 mL, independently of the weight). Thirty minutes before inducing the lesions, the animals received calein D (suspended in 0.05% Tween 80), carbenoxolone (dissolved in water) or the vehicle only (control group). Two hours after the ethanol procedure, the animals were sacrificed in a CO_2_ chamber and their stomachs were immediately dissected and filled with 2% formalin. These organs were opened 5 min later along the greater curvature and the area of the lesions was measured in mm^2^, constituting the ulcer index. The percentage of gastroprotection was calculated accordingly [23]. 

### 4.5. Ethanol-Induced Gastric Mucosal Lesions in l-NAME Pretreated Mice

To investigate the involvement of endogenous NO in the protective effect of calein D, l-NAME (70 mg/kg, dissolved in saline solution) was intraperitoneally administered to three groups of animals, each of which was subsequently given one of three oral treatments (0.05% Tween 80, 30 mg/kg calein D or 100 mg/kg carbenoxolone). After 30 min, all animals received 0.2 mL of ethanol. Two hours later, the animals were sacrificed in a CO_2_ chamber, their stomachs were removed, and the values of the ulcer index were determined. A control group untreated with l-NAME was included [12].

### 4.6. Ethanol-Induced Gastric Mucosal Lesions in Indomethacin-Pretreated Mice

To evaluate the participation of endogenous prostaglandins in the gastroprotective effect of calein D, indomethacin was administered subcutaneously (10 mg/kg, dissolved in NaHCO_3_ 5mM) to three groups of animals, which were then left in their cages for 75 min. Upon completion of this time, they were treated with calein D (30 mg/kg), carbenoxolone (100 mg/kg) or the vehicle. After 30 min, 0.2 mL of ethanol was applied to all animals. Two hours later, the animals were sacrificed in a CO_2_ chamber, their stomachs were removed, and the values of the ulcer index were determined. A control group untreated with indomethacin was included [12].

### 4.7. Ethanol-Induced Gastric Mucosal Lesions in NEM Pretreated Mice

To examine the possible contribution of endogenous sulfhydryl groups to the gastroprotective effect of calein D, three groups were administered 10 mg/kg of *N*-ethylmaleimide (dissolved in saline solution) 30 min before applying one of the three oral treatments (0.05% Tween 80, 30 mg/kg calein D or 100 mg/kg carbenoxolone). A control group untreated with *N*-ethylmaleimide was included. All animals received ethanol (0.2 mL) 30 min after these treatments, and 2 h later they were sacrificed in a CO_2_ chamber to determine the ulcer index [12]. 

### 4.8. Statistics

Data are expressed as the mean ± SEM (*n* = 7). Statistical significance between treatments was analyzed by the Kruskal–Wallis test, followed by Dunn’s multiple comparison, with * *p* ≤ 0.05 considered as significant.

## 5. Conclusions

Scientific evidence is herein provided for the first time that calein D has gastroprotective activity, emphasizing the possible advantage of using sesquiterpene lactones with a germacrane skeleton to develop drugs for ulcer therapy. Further research is necessary to establish the mechanism of calein D, which is not related to nitric oxide, prostaglandins or sulfhydryl groups. Given a previous study finding that 2,3-epoxyjuanislamin and calealactone B (two other sesquiterpene lactones with a germacrane skeleton) can activate the Nrf2 system, the same result is likely for calein D. We will explore this hypothesis in the near future.
